# Corrosion Characteristics of Copper-Added Austempered Gray Cast Iron (AGCI)

**DOI:** 10.3390/ma12030503

**Published:** 2019-02-06

**Authors:** Asiful H. Seikh, Amit Sarkar, Jitendra Kumar Singh, Sohail M. A. Khan Mohammed, Nabeel Alharthi, Manojit Ghosh

**Affiliations:** 1 Centre of Excellence for Research in Engineering Materials, King Saud University, Riyadh 11421, Saudi Arabia; alharthy@ksu.edu.sa; 2 Department of Metallurgical and Materials Engineering, Jadavpur University, Kolkata 700032, India; amitsarkar553@gmail.com; 3 Department of Architectural Engineering, Hanyang University, Ansan 15588, Korea; 4 Department of Mechanical and Industrial Engineering, Ryerson University, Toronto, ON M5B 2K3, Canada; sohailmazher5@gmail.com; 5 Mechanical Engineering Department, College of Engineering, King Saud University, Riyadh 11421, Saudi Arabia; 6 Department of Metallurgy and Materials Engineering, Indian Institute of Engineering Science and Technology, Shibpur 711103, India; manojit_ghosh1@rediffmail.com

**Keywords:** austempered gray cast iron, austempering temperature, microstructure, potentiodynamic polarization, electrochemical impedance spectroscopy

## Abstract

The aim of this investigation was to assess the corrosion behavior of gray cast iron (GCI) alloyed with copper. Alloyed GCI specimens were austempered isothermally at varying temperatures. After austenitizing at 927 °C, the samples were austempered at different temperatures ranging from 260 to 385 °C with an interval of 25 °C for 60 min. As a result, these samples developed an ausferrite matrix with different percentages of austenite. The resulting microstructures were evaluated and characterized by optical microscope (OM), scanning electron microscope (SEM), and X-ray diffraction (XRD). The corrosion characteristics were determined using potentiodynamic polarization tests and electrochemical impedance spectroscopy (EIS) of these samples. These tests were carried out in a medium of 0.5 M H_2_SO_4_ and 3.5% NaCl solution. It was observed from the potentiodynamic polarization results that with increasing austempering temperature, the corrosion rate decreased. All results of the EIS were in accordance with a constant phase element (CPE) model. It was found that with an increase in austempering temperature, the polarization resistance (R_p_) increased. The austenite content was also found to influence the corrosion behavior of the austempered gray cast iron (AGCI).

## 1. Introduction

Gray cast iron (GCI) is a potential engineering material, which has a diverse range of applications including use in sophisticated automotive parts [1]. The wide applications of GCI are possible due to its unique properties such as good thermal conductivity, relatively low melting temperature, high damping capacity, and excellent castability [1,2]. The damage of the GCI components at the exterior parts through electrochemical corrosion has been the predominant restricting mechanism against enhancing its life span [3]. The presence of graphitization is a distinguishing feature of the deterioration properties of GCI [3,4]. Attempts have been made to combat the problem of corrosion with the help of alloying additions with the aim of modifying the microstructure from ferrite to fine pearlite. It is also pertinent to mention that Si plays an important role in controlling the corrosion behavior of GCI: The higher the Si content, the higher the corrosion resistance [5]. Additionally, it is well known that single-phase microstructures like austenite, ferrite, and martensite perform better in corrosive media compared to two-phase mixtures like bainite, pearlite, and tempered martensite [6]. 

Several researchers [7,8,9,10] have tried to assess the effect of heat treatment and composition on the microstructure and corrosion behavior of austempered ductile iron (ADI). Prasanna et al. [7] and Banerjee et al. [8] studied the effect of the austempering treatment on the microstructure and corrosion properties of ductile iron. They found that both mechanical properties and corrosion resistance were enhanced due to the austempering of cast iron. Afolabi et al. [9] observed that the austempering temperature and time influenced the microstructure of the ductile iron, and thus its corrosion behavior was affected by the compositional structures. Hsu and Chen [10] concluded that the enhancement of corrosion resistance in ADI was due to the presence of retained austenite as a result of austempering. Similar studies with GCI are also common where the corrosion resistance and the mechanical properties were improved dramatically by tailoring the heat treatment pattern (tempering, austempering, and quenching [11,12,13,14]) and by alloy additions [8]. Further improvement of the mechanical properties, compared to those of conventional GCI, was observed in austempered gray cast iron (AGCI) due to the formation of a matrix of ausferritic structures (ferrite and stabilized austenite) or bainitic ferrite during austempering [12,15]. Thus, the domain of applicability of AGCI is even wider than that of GCI due to its favorable combination of enhanced mechanical properties [11,12,13,14,15] and improved wear characteristics [15,16,17]. The present literature, however, is lacking in reporting the corrosion behavior of AGCI, although a lot of work can be found on testing the corrosion behavior of ADI [7,8,9,10]. 

The above scientific observations indicated the necessity of the present investigation into the effect of austempering temperatures on the microstructure and corrosion behaviors of copper-alloyed AGCI, in order to establish a correlation between them. 

## 2. Materials and Methods

### 2.1. Sample Preparation

**Figure 1:**
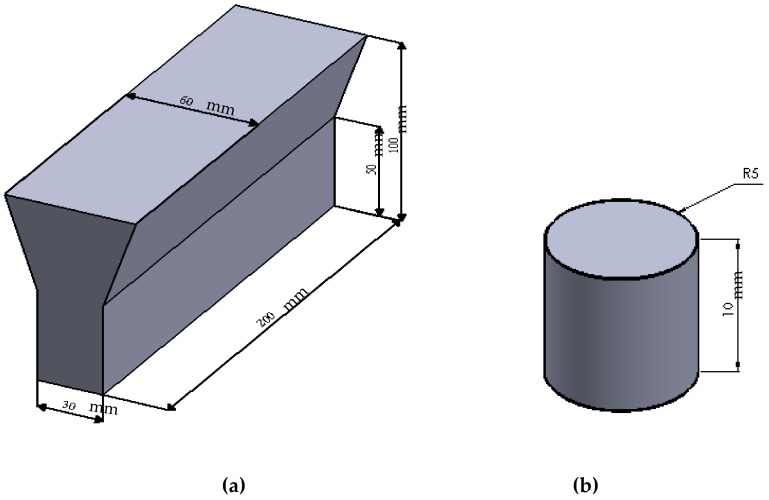
(**a**) Dimension (mm) of the Y-block casting and (**b**) schematic of the corrosion test piece (Φ 10 mm × 10 mm).

Samples of GCI were prepared from cupola melts in a production foundry. The molten metal, at a temperature of 1420 °C was inoculated with 0.25 wt. % of FeSi-based inoculants in the cupola. During tapping, 0.5% Cu pieces of electrolyte grade were added to the metal stream for the sake of alloying. The specimens were cast in the form of standard 30-mm Y-shaped blocks in sand molds as shown in Figure 1a. Corrosion test coupons (Figure 1b) of suitable size (Φ 10 mm × 10 mm) were machined from the as-cast Y blocks. The uniform distribution of fine type-A graphite flakes is promoted by the inoculants during solidification [18]. Cu is soluble in austenite and increases the hardness, strength, corrosion resistance, and transformation time for the austempering process [19,20]. Cu has been accepted as an affordable alloying element for several engineering applications. As a result, the replacement of expensive Ni by Cu may become more prevalent. The final chemical composition (wt. %) of GCI was determined using a spectroscopy spark analyzer as shown in Table 1.

### 2.2. Heat Treatment of Samples

**Figure 2:**
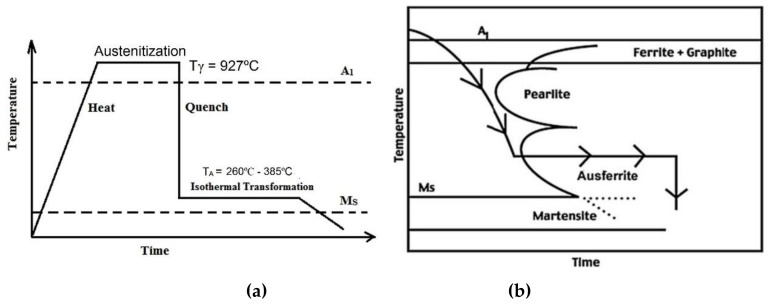
Schematic diagram of (**a**) heat treatment schedule for austempering and (**b**) CCT diagram for the proposed composition.

The samples were initially heated to an austenitizing temperature (Tγ = 927 °C) and held for 60 min in order to develop a fully austenitic structure (γ). The samples were then rapidly cooled in a molten salt bath comprising 53% KNO_3_, 40% NaNO_2_, and 7% NaNO_3_ at six different austempering temperatures (T_A_), 260, 285, 310, 335, 360, and 385 °C, for 60 min followed by air cooling to complete the phase transformation. Figure 2a schematically represents the entire heat treatment schedule for the austempering process and Figure 2b is the corresponding continuous cooling transformation (CCT) diagram. 

### 2.3. Metallography and X-ray Diffraction (XRD) 

Samples were prepared for metallographic observation using standard polishing techniques. Moreover, the samples were etched using a 2% nital solution for observation under a scanning electron microscope (SEM, JSM 6360, Jeol techniques, Tokyo, Japan). The volume fractions of austenite were calculated by X-ray diffraction (XRD,) analysis as described by Dasgupta et al. [21]. The XRD data were collected using a Rigaku, Ultima III diffractometer (Japan) with a monochromatic copper Fe-Kα radiation (1.54 Å) at 40 kV and 30 mA. Scanning was done at a rate of 1°/min from 30 to 90° to observe the peaks, which were later analyzed using Jade 7 software (7.1.08). The peak positions were analyzed for the (111), (220), and (311) planes of austenite (FCC) and the (110), (200), and (211) planes of ferrite (BCC). The carbon content in austenite (C_γ_) at various austempering temperatures was calculated using the following equation:

(1) $$C_{\gamma }=\frac{a_{\gamma }-3.548}{0.044},$$

where *a_γ_* is the lattice parameter calculated from the angular position of the austenite peak [22].

### 2.4. Electrochemical (Corrosion) Test

The heat-treated samples were subjected to electrochemical measurements in a 0.5 M H_2_SO_4_ and 3.5% NaCl solution at 25 °C (±2 °C). The electrochemical studies were performed in a triplicate set of samples to obtain reproducible results. A cell composed of three electrodes was created, including a graphite one, which acts as a counter electrode; a saturated calomel electrode (SCE), which acts as a reference electrode; and the GCI sample which acts as the working electrode (WE), for potentiodynamic polarization and electrochemical impedance spectroscopy (EIS) measurements. The WE area was fixed at 1 cm^2^. Prior to the tests, the samples were ground and polished using SiC papers of 2500 grit size and rinsed in deionized water followed by immersion in the solution for 30 min in order to stabilize the open circuit potential value. The potentiodynamic polarization tests were carried out from −1.0 to +1.0 V at a scan rate of 1 mV/s, whereas, EIS tests were performed over a frequency ranging from 100 kHz to 0.01 Hz.

## 3. Results and Discussion

### 3.1. Microstructure and XRD Analysis

**Figure 3:**
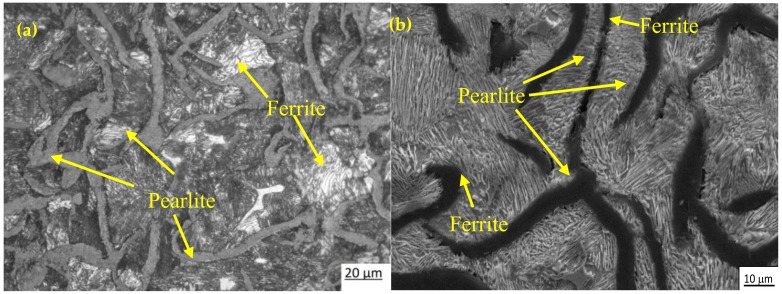
Micrographs of as-cast specimens: (**a**) optical micrograph and (**b**) SEM image.

**Figure 4:**
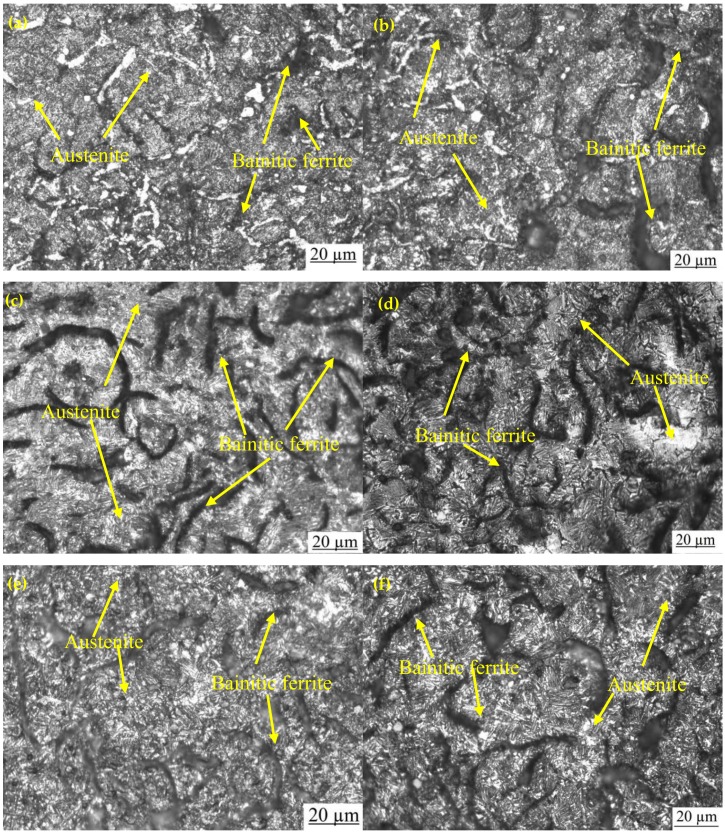
Optical micrographs of samples austempered for 60 min at (**a**) 260 °C, (**b**) 285 °C, (**c**) 310 °C, (**d**) 335 °C, (**e**) 360 °C, and (**f**) 385 °C.

**Figure 5:**
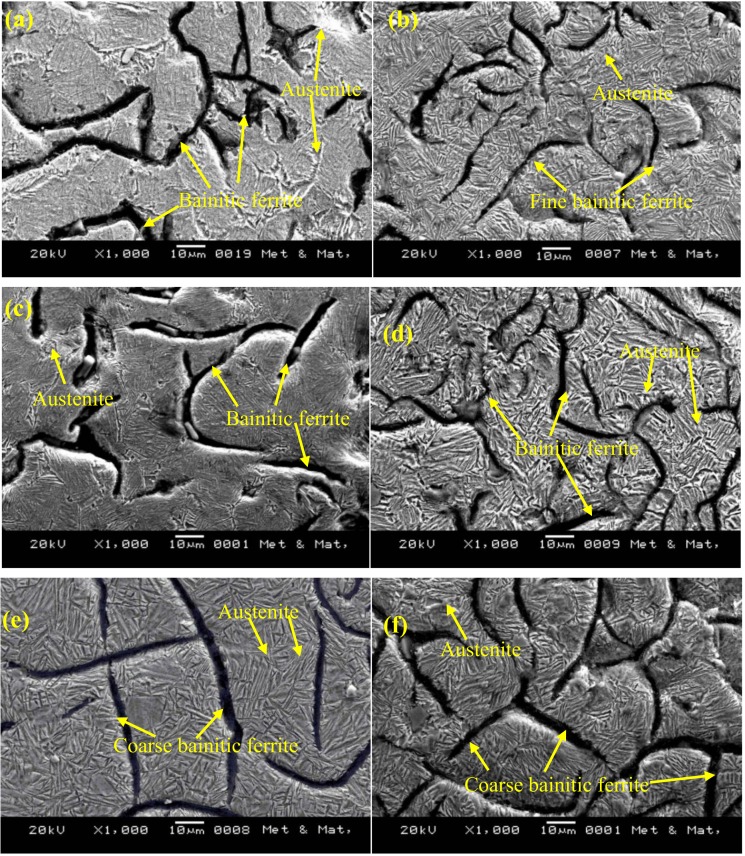
SEM images of samples austempered for 60 min at (**a**) 260 °C, (**b**) 285 °C, (**c**) 310 °C, (**d**) 335 °C, (**e**) 360 °C, and (**f**) 385 °C.

Figure 3a,b shows the optical and SEM microstructures of the as-cast gray iron sample, respectively. The matrix of as-cast gray iron is primarily composed of pearlite besides some randomly distributed ferrite. The as-cast specimens were austempered for 60 min and the resulting changes in the microstructure are presented in Figure 4a–f and Figure 5a–f using an optical microscope (OM) and SEM, respectively. The dark, etched needle-like structures represent bainitic ferrite, while the brighter ones represent a mixture of austenite and bainitic ferrite. We can see that the effect of the austempering temperature on the microstructure of austempered irons was significant. It was observed that at lower temperatures (i.e., 260–285 °C), very fine needles of bainitic ferrite and austenite were formed and the volume fraction of ferrite was larger. As the austempering temperature increased, the needles of the bainitic ferrite were coarsened along with an increase in the austenite content. Similar observations in ADI were earlier reported by Patutunda et al. [23] and Yang et al. [24].

**Figure 6:**
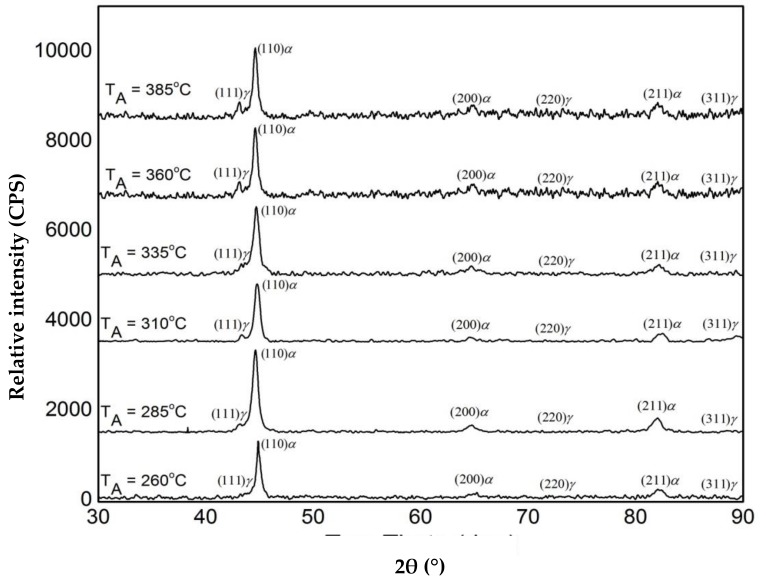
XRD phase analysis of austempered gray cast iron (AGCI) for different austempering temperatures held for 60 min (**a**) 260 °C, (**b**) 285 °C, (**c**) 310 °C, (**d**) 335 °C, (**e**) 360 °C, and (**f**) 385 °C.

**Figure 7:**
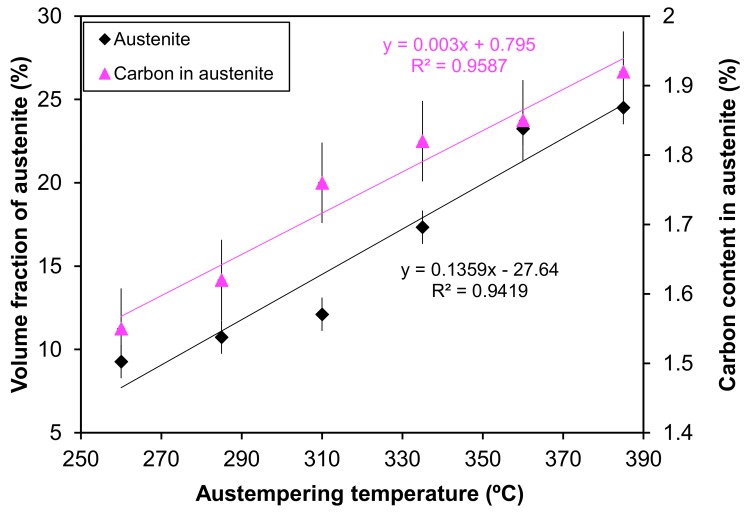
The influence of different austempering temperatures on the volume fraction of austenite and the carbon content of austenite.

Figure 6 presents the quantitative analysis of the XRD pattern. It is evident from the figure that the austempering temperature has a significant effect on the XRD patterns. It was seen that with changing heat treatment temperature, the amount of austenite was changed. The phases detected include ferrite and austenite. Figure 7 shows the volume fraction of austenite and the carbon content of austenite in the AGCI samples as a function of different austempering temperatures. The volume faction of austenite was calculated by Jade7 software built in the XRD. It may be noted from Figure 7 that the austenite content increases with an increase in the austempering temperature. Greater supercooling at a lower austempering temperature resulted in finer ferrite and austenite as also reported by Patutunda et al. [23]. It is well known that the transformation reaction is more likely to be controlled by the nucleation process rather than growth [23,24]. During the process, it is necessary that the carbon must diffuse into austenite through the ferrite zone. At higher austempering temperatures a quite contrasting mechanism prevails due to lower supercooling which makes the nucleation of ferrite slower. This leads to the stabilization of more austenite in addition to incrementing the rate of diffusion of carbon which leads to the formation of coarse ferrite. Thus, the volume fraction of austenite increases with the increase in austempering temperature.

### 3.2. Electrochemical Behavior of As-Cast Gray Iron and AGCI in Different Solutions

**Figure 8:**
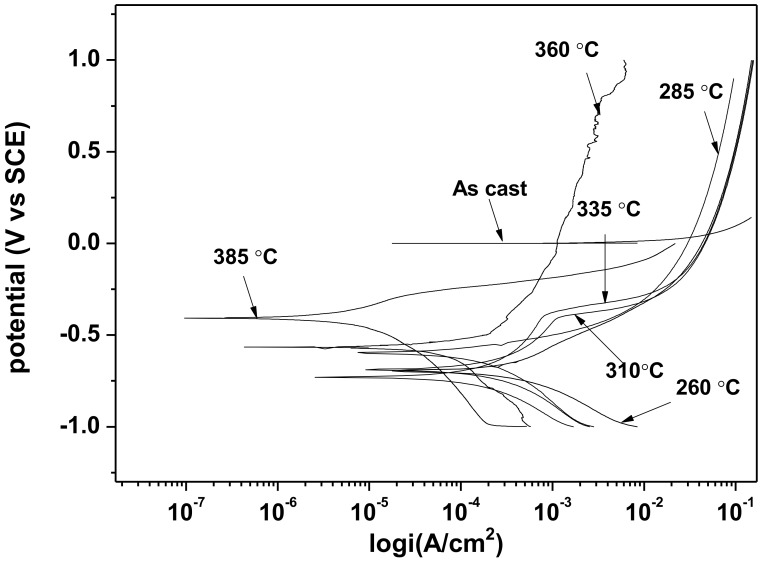
Potentiodynamic polarization curves in 3.5% NaCl solution.

**Figure 9:**
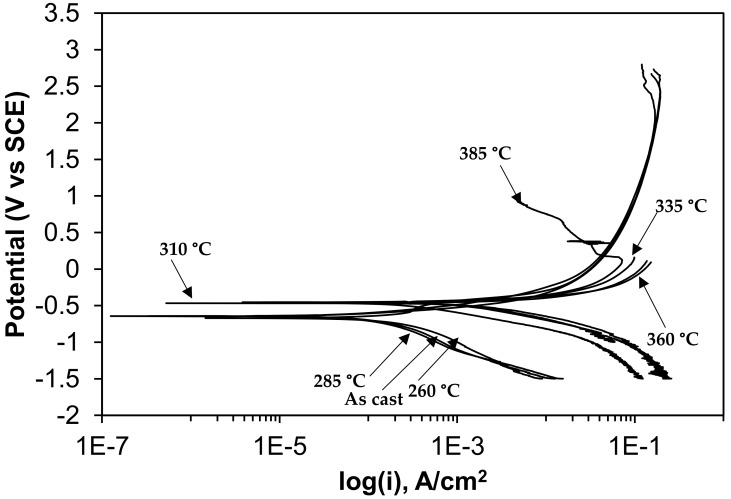
Potentiodynamic polarization curves in 0.5 M H_2_SO_4_ solution.

The results of potentiodynamic polarization studies of the as-cast gray iron and the AGCI are shown in Figure 8 and Figure 9. The corrosion of iron in neutral 3.5% NaCl solution occurs according to the following equations,

Anodic reaction:

Fe → Fe^2+^ + 2e, (2)

Cathodic reaction:

O_2_ + 2H_2_O + 4e → 4OH^−^. (3)

When iron is in contact with dilute sulfuric acid (0.5 M H_2_SO_4_), an immediate attack on the metal takes place with the formation of hydrogen gas and ferrous ions, as shown in Equations (4) and (5).

Anodic reaction:

Fe → Fe^2+^ + 2e^−^. (4)

Cathodic reaction:

2H^+^ + 2e^−^ → H_2_. (5)

The electrochemical parameters are extracted after the extrapolation of the potentiodynamic plots in a Tafel slope. From Figure 7 it is revealed that with increasing austempering temperature, the percentage of austenite increased while the corrosion current density (I_corr_) decreased and the corrosion potential (E_corr_) shifted to the cathodic side (Table 2 and Table 3). Austenite acts as an anode and ferrite acts as a cathode. The galvanic corrosion is proportional to the cathodic/anodic area. Consequently, with increasing temperature, the austenite percentage increases with a simultaneous decrease in the ferrite percentage. Thus, due to the microstructural homogeneities, distinct localized anodic and cathodic microstructural areas develop, which act as micro-electrochemical cells in the presence of an electrolyte. Thus, the galvanic corrosion decreases in both solutions. From Table 2 and Table 3, it can be seen that among the two corrosive mediums, 1 N H_2_SO_4_ is more corrosive in all cases.

**Figure 10:**
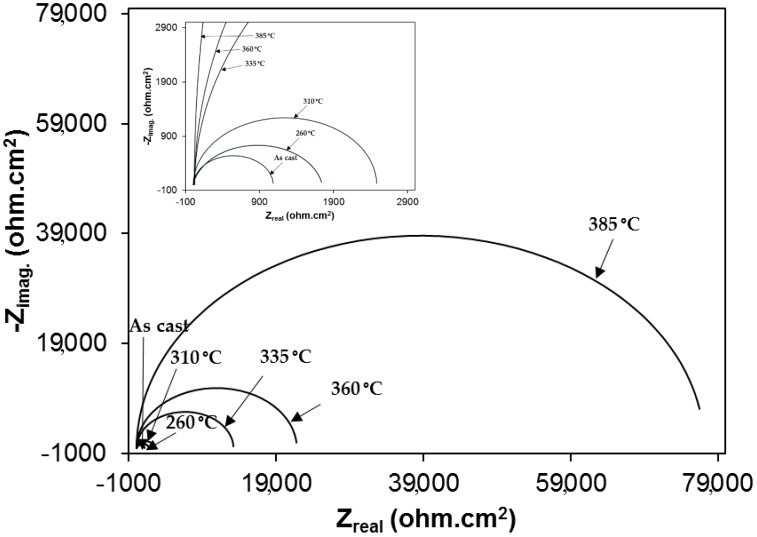
Electrochemical impedance spectroscopy (Nyquist plot) in 3.5% NaCl solution.

**Figure 11:**
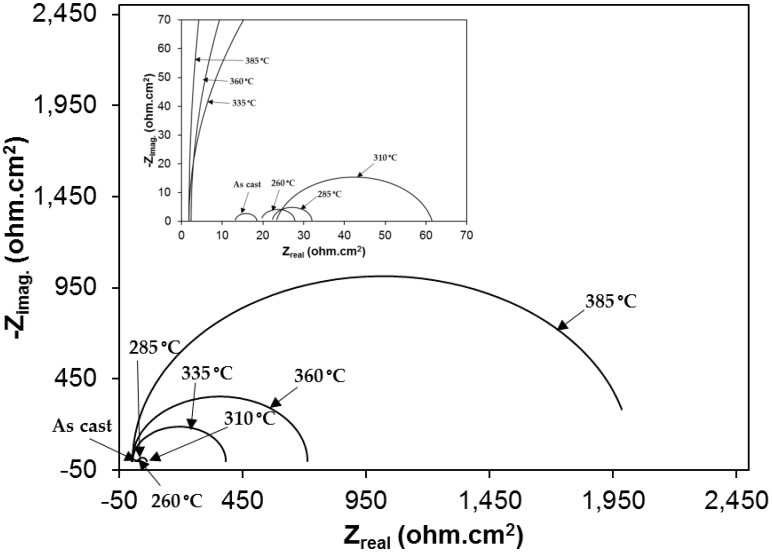

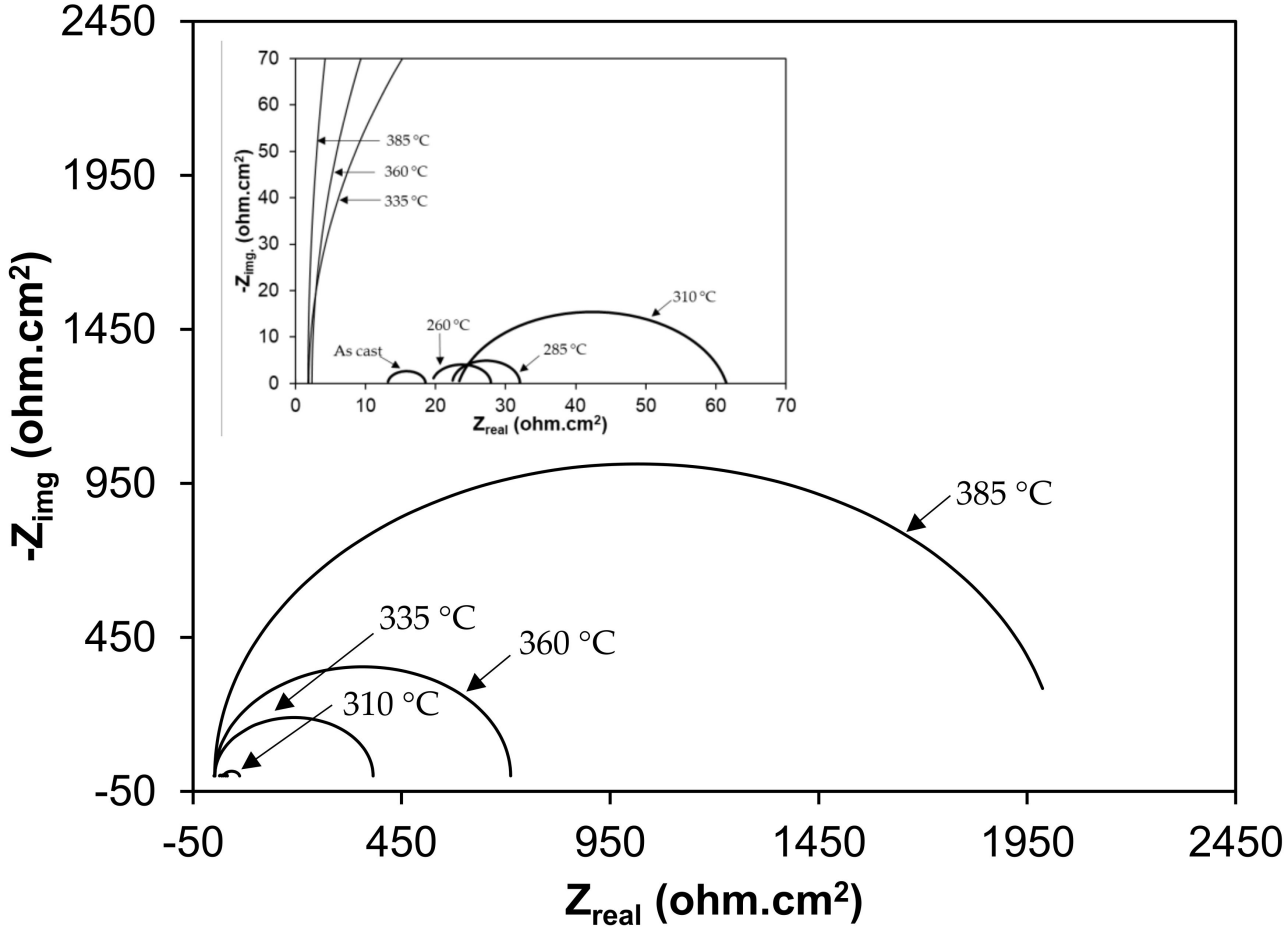
Electrochemical impedance spectroscopy (Nyquist plot) in 1 N H_2_SO_4_ solution.

**Figure 12:**
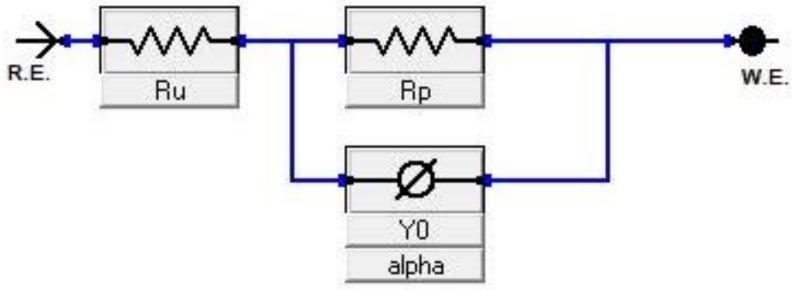
Equivalent circuit of electrochemical impedance spectroscopy (EIS).

Nyquist plots of samples exposed to 3.5% NaCl and 0.5 M H_2_SO_4_ solutions are shown in the Figure 10 and Figure 11, respectively. All plots show a depressed semicircle pattern in the whole frequency range, indicating that only one time constant exists between the interface of the solid electrode and the solution. Due to the low impedance value at the lower austempering temperature, the Nyquist plots become suppressed. The corresponding plots are shown in the insets of Figure 10 and Figure 11 for exposure to 3.5% NaCl and 0.5 M H_2_SO_4_ solutions, respectively. All the EIS data match well in a constant phase element (CPE) model. In a CPE model, R_u_ is the solution resistance, R_p_ is the polarization resistance, and Y_o_ is the admittance. The inserted equivalent circuit shown in Figure 12 was used to fit the EIS data, and the fitted polarization resistance (R_p_) data are shown in Table 4 and Table 5 for 3.5% NaCl and 0.5 M H_2_SO_4_ solution, respectively. It is known that the diameter of the Nyquist plot represents the R_p_. It is also well known that the R_p_ is inversely proportional to the corrosion rate. With increasing austempering temperature, the diameter of the Nyquist plot increases with a consequent increase in the R_p_. It is also seen from Table 4 and Table 5 with the error bar (maximum ±7%) that the R_p_ is higher in 3.5% NaCl solution (Table 4) than it is in 0.5 M H_2_SO_4_ solution (Table 5) for all cases.

### 3.3. Effect of Austenite Content on Corrosion Behavior

**Figure 13:**
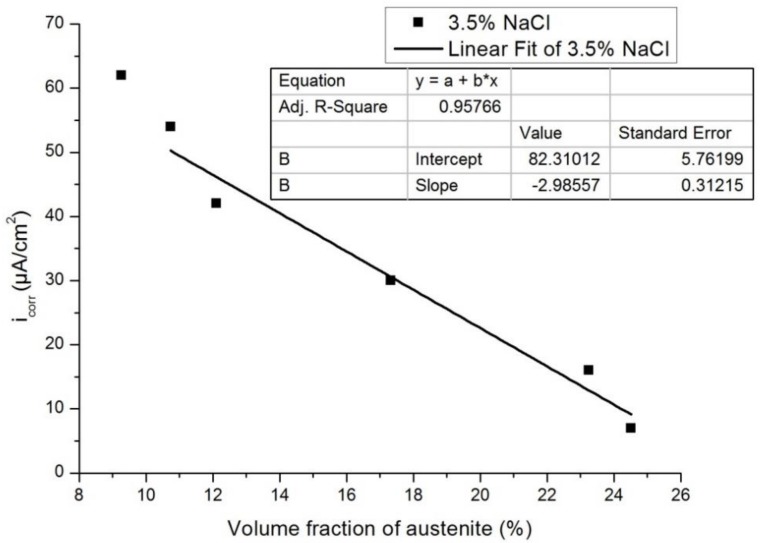
Influence of the volume fraction of the austenite on corrosion rate in 3.5% NaCl.

**Figure 14:**
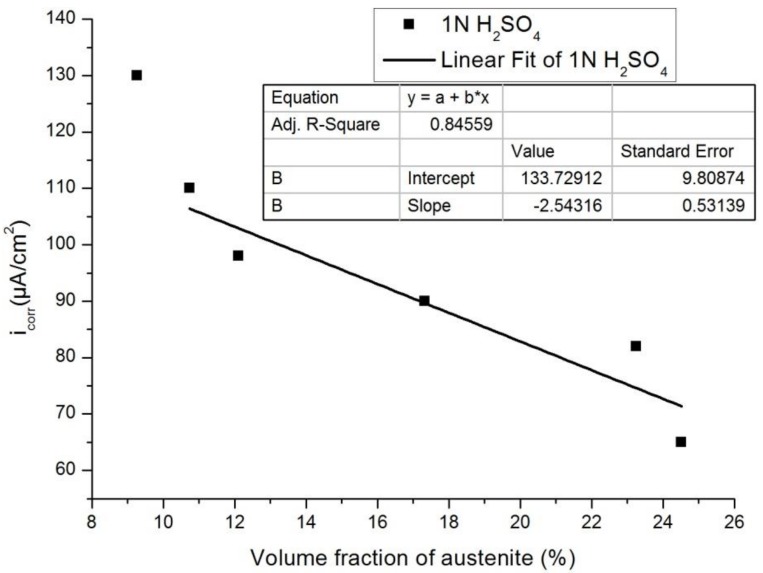
Influence of the volume fraction of the austenite on the corrosion rate in 0.5 M H_2_SO_4_.

Figure 13 and Figure 14 show the plots of I_corr_ obtained from the potentiodynamic polarization diagram against the volume fraction of the austenite for 3.5% NaCl and 1 N H_2_SO_4_, respectively. It was observed that with the increasing volume fraction of austenite, the corrosion rate decreased linearly to a sufficient extent in both cases. The linear fit regression value was 0.95 for 3.5% NaCl solution (Figure 13) and 0.84 for 1N H_2_SO_4_ solution (Figure 14). A regression value close to 1 means the corrosion rate changes linearly with increasing austenite content.

### 3.4. Microstructure after Corrosion

#### 3.4.1. Optical Images after Corrosion in 3.5% NaCl Solution

**Figure 15:**
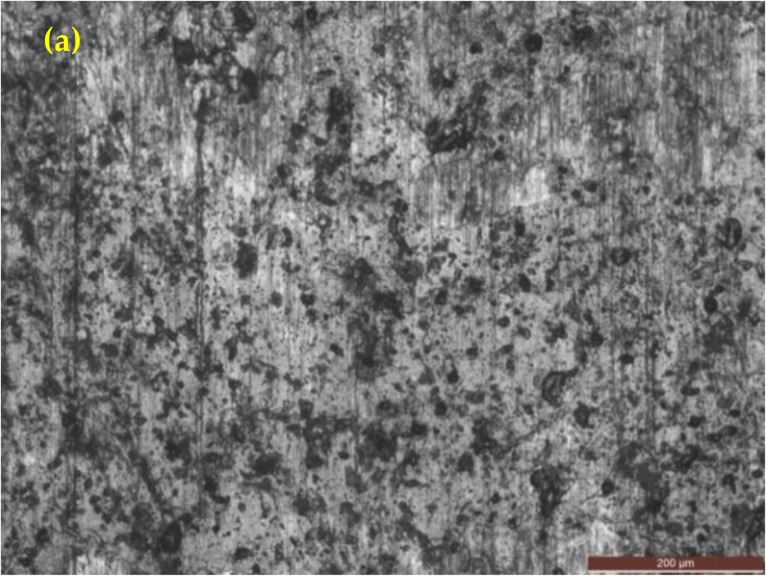

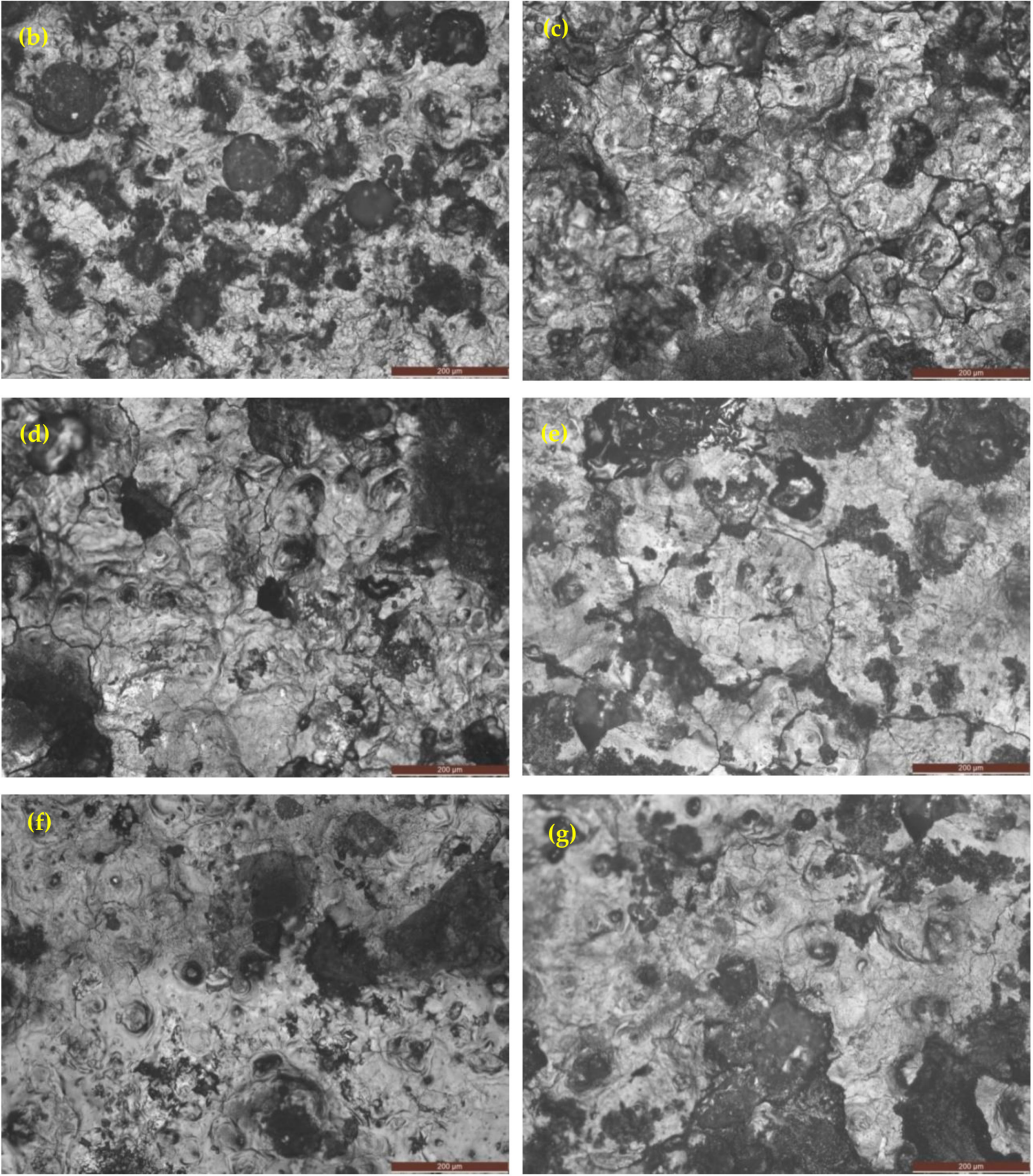
Optical images of iron samples after a potentiodynamic polarization test in 3.5% NaCl solution. (**a**) As-cast gray iron; and AGCI at (**b**) Tγ = 927 °C, T_A_ = 260 °C; (**c**) Tγ = 927 °C, T_A_ = 285 °C; (**d**) Tγ = 927 °C, T_A_ = 310 °C; (**e**) Tγ = 927 °C, T_A_ = 335 °C; (**f**) Tγ = 927 °C, T_A_ = 360 °C; and (**g**) Tγ = 927 °C, T_A_ = 385 °C.

Figure 15 shows the OM of as-cast gray iron and AGCI samples dipped in 3.5% NaCl solution. The corrosion products consist of compact structures. It is seen that compactness increases with increasing austempering temperature. It was also observed that in optical images of as-cast gray iron (Figure 15a) with a lower austempering temperature (260 °C and 285 °C), an exfoliation type pattern was present with smaller flake graphite. With increasing austempering temperature (310 °C and above), the exfoliation type pattern disappeared with bigger flake graphite. In addition, intergranular type corrosion was observed in the 3.5% NaCl solution. It is also seen that, in as-cast gray iron at a low austempering temperature, more pitting was seen. However, with increasing austempering temperature the pitting density decreased.

#### 3.4.2. Optical Images after Corrosion in 0.5 M H_2_SO_4_ Solution

**Figure 16:**
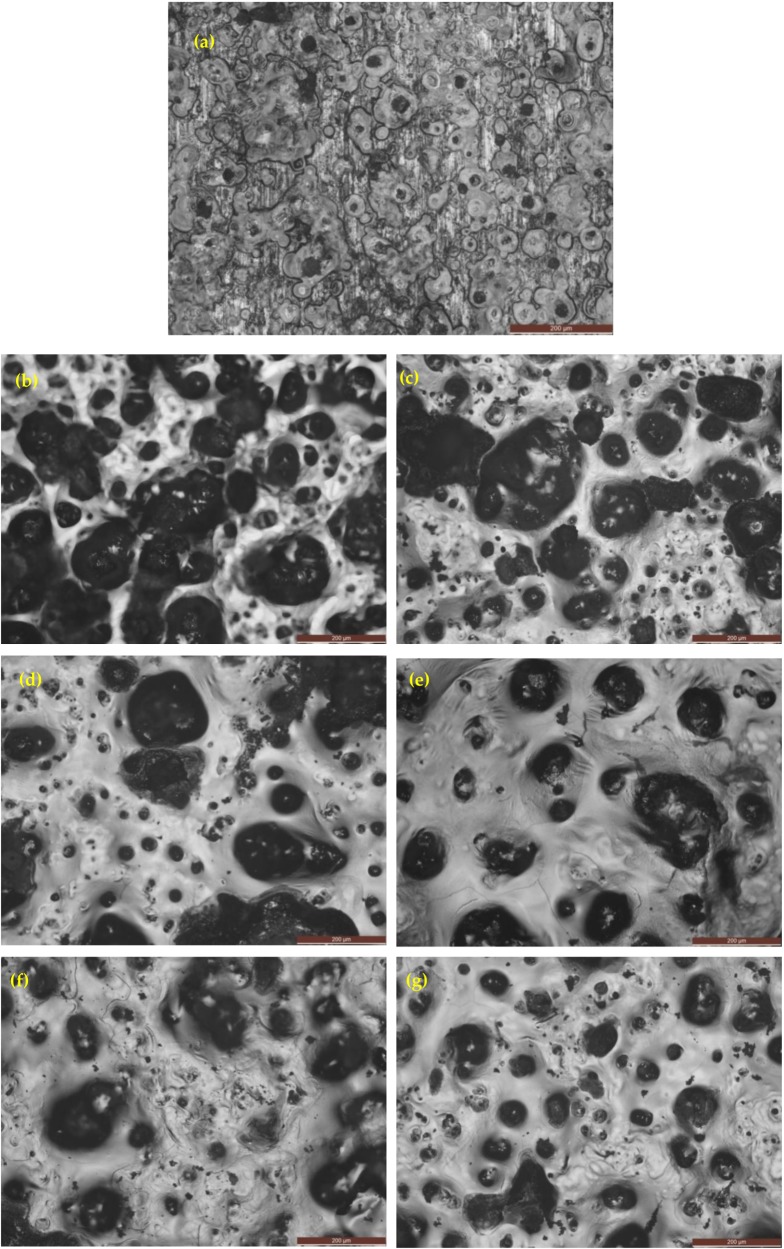
Optical images of iron samples after a potentiodynamic polarization test in 1 N H_2_SO_4_ solution. (**a**) As-cast gray iron; and AGCI at (**b**) Tγ = 927 °C, T_A_ = 260 °C; (**c**) Tγ = 927 °C, T_A_ = 285 °C; (**d**) Tγ = 927 °C, T_A_ = 310 °C; (**e**) Tγ = 927 °C, T_A_ = 335 °C; (**f**) Tγ = 927 °C, T_A_ = 360 °C; and (**g**) Tγ = 927 °C, T_A_ = 385 °C.

Figure 16 shows the optical images (as-cast gray iron and AGCI) of corrosion products in 0.5 M H_2_SO_4_ solution. It is seen that pitting formation decreased, with increasing austempering temperature. While with increasing austempering temperature, more metastable pits were formed. So it can be concluded that with increasing austempering temperature, pit formation gradually reduced. At the same temperature, compared to 3.5% NaCl solution, pitting density, radius of pits, and the flake graphite was larger in the case of 0.5 M H_2_SO_4_ solution_._ It is also seen that more pits are formed in 1 N H_2_SO_4_ than in 3.5% NaCl solution.

## 4. Conclusions

The following conclusions can be drawn from the present investigation:

(a) The microstructure of AGCI consists of special bainitic ferrite (α) and high-carbon austenite (Y) which prevents corrosion. Thus, the corrosion-resistance susceptibility of AGCI is higher than that of as-cast gray iron.

(b) At higher austempering temperatures, the volume fraction of austenite increases with a consequent decrease in the corrosion rate. 

(c) In the ausferrite matrix, the corrosion rate depends on the austenite content. An increase in the austenite content results in a decrease in the corrosion rate.

(d) Due to an increase in hydrogen generating reactions, 1 N H_2_SO_4_ is more corrosive than 3.5% NaCl during exposure.

## Figures and Tables

**Table 1:** Chemical composition of GCI (wt. %).

Element	C	Si	Mn	P	S	Cu	Fe
Composition	3.46	2.27	0.53	0.019	0.01	0.50	Bal.

**Table 2:** Potentiodynamic polarization results in 3.5% NaCl.

Sample Condition	I_corr_ (μA/cm^2^)	E_corr_ (V)
Austempering Temperature (°C)	-	-
As-cast gray iron	70	–0.80
927 °C–260 °C	62	–0.75
927 °C–285 °C	54	–0.70
927 °C–310 °C	42	–0.62
927 °C–335 °C	30	–0.50
927 °C–360 °C	16	–0.45
927 °C–385 °C	7	–0.40

**Table 3:** Potentiodynamic polarization results in 1 N H_2_SO_4_.

Sample Condition	I_corr_ (µA/cm^2^)	E_corr_ (V)
Austempering Temperature (°C)	-	-
As-cast gray iron	150	–0.60
927 °C–260 °C	130	–0.55
927 °C–285 °C	110	–0.50
927 °C–310 °C	98	–0.48
927 °C–335 °C	90	–0.42
927 °C–360 °C	82	–0.38
927 °C–385 °C	65	–0.36

**Table 4:** Electrochemical impedance spectroscopy results in 3.5% NaCl solution.

Sample Condition	R_p_ (ohm.cm^2^)
Austempering Temperature (°C)	-
As-cast gray iron	930 (± 46)
927 °C–260 °C	1850 (± 91)
927 °C–310 °C	2550 (± 102)
927 °C–335 °C	15,000 (± 450)
927 °C–360 °C	20,000 (± 1100)
927 °C–385 °C	80,000 (± 1600)

**Table 5:** Electrochemical impedance spectroscopy results in 0.5 M H_2_SO_4_ solution.

Sample Condition	R_p_ (ohm.cm^2^)
Austempering Temperature (°C)	-
As-cast gray iron	20 (± 1)
927 °C–260 °C	28 (± 1)
927 °C–285 °C	32 (± 2)
927 °C–310 °C	62 (± 2)
927 °C–335 °C	450 (± 13)
927 °C–360 °C	700 (± 28)
927 °C–385 °C	2000 (± 85)
